# Tobacco Treatment Outcomes for Hospital Patients With and Without Mental Health Diagnoses

**DOI:** 10.3389/fpsyt.2022.853001

**Published:** 2022-05-26

**Authors:** Brandon T. Sanford, Benjamin A. Toll, Amanda M. Palmer, Madeline G. Foster, K. Michael Cummings, Stephanie Stansell, Alana M. Rojewski

**Affiliations:** ^1^Department of Public Health Sciences, Medical University of South Carolina, Charleston, SC, United States; ^2^Hollings Cancer Center, Medical University of South Carolina, Charleston, SC, United States; ^3^Department of Psychiatry and Behavioral Sciences, Medical University of South Carolina, Charleston, SC, United States

**Keywords:** smoking cessation, mental health, tobacco, inpatient, opt-out

## Abstract

**Background:**

The prevalence of mental health conditions is higher in cigarette smokers than nonsmokers. However, those with diagnosed mental health disorders are understudied within general inpatient hospital settings. This study seeks to evaluate how having a mental health diagnosis influences response to a brief opt-out inpatient tobacco treatment intervention.

**Methods:**

Data included 4,153 admitted patients who completed a tobacco treatment visit. Post-discharge self-reported abstinence was obtained via response to an automated call 1-month after discharge. Mental health co-morbidities were assessed by reviewing electronic medical records. Logistic regression was used to assess associations between having a mental health diagnosis and patients' smoking history, interest in quitting smoking, and post-discharge abstinence.

**Results:**

Overall 34.1% of patients were diagnosed with mental health disorders, most commonly depression or substance use disorders. Patients with a diagnosed mental health disorder were more likely to report a history of long-term heavy smoking and were less likely to express an interesting in remaining abstinent from smoking after hospitalization. An intent-to-treat analysis using logistic regression analysis found lower rates of self-reported smoking abstinence in those with a mental health disorder compared to those without (9 vs. 13.2%, *p* < 0.001).

**Conclusions:**

Patients with a history of mental health diagnoses, such as depression or substance use disorders, was associated with lower rates of smoking abstinence in patients after hospitalization. Hospital based opt-out smoking cessation programs have shown to be generally effective and efficient. However, certain subpopulations may require tailored intervention in order to improve treatment outcomes. Future research is needed to develop brief, effective tobacco treatment for hospital patients with comorbid mental health diagnoses.

## Introduction

According to the U.S. Department of Health and Human Services, 14 out of every 100 U.S. adults smokes cigarettes (1) which equates to approximately 34.1 million Americans at the time of this writing. However, tobacco use is not evenly distributed across the population. Those with diagnosed mental health or substance use disorders are nearly three times as likely to smoke compared to those without [41%; (2, 3)]. Rates of smoking among those diagnosed with schizophrenia have been shown to be ~62% (4, 5). While rates of smoking have been generally declining at a population level, this is not true of those with psychiatric comorbidity (6) across domains of mental health problems. Approximately 20% of US adults have a current mental health problem (7), and this group has been estimated to consume between 44 and 50% of all cigarettes in the United States (8). Unfortunately, those with mental health conditions are significantly less likely to quit smoking (9) and psychiatric patients' tobacco dependence is rarely addressed in routine clinical practice (10, 11), despite efforts focused on tobacco cessation being associated with improved mental health (12, 13). This is especially important as those with serious mental illness suffer premature mortality rates predominately caused by cardiovascular and respiratory diseases (14, 15).

One initiative to address hospital-based smoking cessation rates at a population level is that of brief opportunistic opt-out interventions where treatment is provided as standard procedure for all patients who smoke rather than by patient request (16). This type of approach address the needs of underserved populations who may not otherwise have access to tobacco treatment interventions. However, there remains a dearth of information regarding the relative effectiveness of these brief interventions with those diagnosed with mental health conditions. Aside from specialty clinical trials focused on patients with mental health conditions, those with mental health diagnoses are usually excluded from general tobacco treatment trials and represent a relatively understudied population within the field of smoking cessation (17).

The present study aims to evaluate the effectiveness of a brief tobacco treatment intervention among a large, hospitalized sample treated by the Tobacco Treatment Program (TTP) at the Medical University of South Carolina. Results from these analyses will provide data regarding the adequacy of services delivered for this population and suggest future directions for improving outcomes among priority populations.

## Methods

### Tobacco Treatment Procedures

Inpatient tobacco treatment services at the hospital are provided via an opt-out system whereby counselors identify and provide services to all patients admitted to the hospital with a reported history of tobacco use. Interventions include a structured assessment interview, brief counseling, and pending smoking cessation pharmacotherapy (e.g., nicotine replacement therapy) orders for physicians to facilitate while inpatient and at discharge. Bedside counseling includes motivational interviewing and practical counseling strategies. Patients are then enrolled in an automated, interactive voice recognition telephone protocol, which calls them at 3, 14, and 30 days following discharge. Smoking status is assessed at these calls, and patients are offered a referral to outpatient counseling or the South Carolina Quitline. Visit notes are recorded in the electronic health record (Epic). This program has demonstrated clinical efficacy in improving treatment outcomes, reducing readmissions, and cutting costs (16).

### Participants

All data were collected as part of routine treatment of general hospital patients within the TTP at Medical University of South Carolina (18). Patients admitted to the inpatient psychiatric hospital were excluded as routine opt-out treatment was not available for the duration of the data collection period.

Chart data were retrieved from patients who were admitted between July 2014 and December 2019. Patients who endorsed cigarette smoking, agreed to the bedside intervention, and accepted enrollment into the interactive voice recognition system were included in the present analysis. Of those identified, patient medical record numbers were used to obtain data on history of mental health conditions. Follow-up data were collected through review of patients' responses to the automated telephone system 30-days following discharge.

### Measures

#### Medical Chart Data

Patients' age, race, and biological sex were obtained from the electronic health record note (TTP encounter) during admission. History of mental health conditions on day of admission was obtained from the electronic health record (i.e., “problem list”) via an internal data request. Mental health diagnoses were then grouped into one of the following broad diagnostic categories: Depression, Anxiety, PTSD, chronic pain, childhood developmental disorder, serious mental illness, personality disorder, alcohol use disorder, or substance use disorder. Serious mental illness was defined as including the following diagnostic categories (1) schizophrenia, (2) bipolar disorder, (3) severe depression with psychotic features, (4) eating disorders.

#### Smoking Characteristics

TTP clinicians asked patients to report on how long they had been smoking, if they smoked daily, how many cigarettes were smoked per day, how soon they smoked after waking, if they use any other tobacco product, and if they live with another person who smokes. Patients were also asked how many times, if any, they tried to quit smoking during the past year. Importance to quit was measured by asking “How important is quitting smoking to you on a scale of 1–5, with 5 being the most important?” Confidence in quitting was measured by asking “How confident are you that you will be able to remain smoke free on a scale of 1–5, with 5 being the most confident?” Finally, patients were asked if they had requested and received a smoking cessation medication during hospitalization.

#### Follow-Up Data

The automated telephone system contacted patients 30-days following discharge. Patients were coded “quit” if they endorsed not smoking for the 7-days prior to the phone call.

### Statistical Procedures

Logistic regressions, utilizing an intent-to-treat approach [ITT; (19)], coding non-responders as smokers, were used to evaluate the impact of mental health diagnoses on smoking behavior and abstinence at follow-up. Correlations were utilized to establish the relationship between mental health diagnoses and quit attempts, importance of quitting, and self-efficacy related to quitting.

## Results

### Patient Characteristics

Chart review identified 4,153 patients who endorsed current cigarette smoking upon admission and completed an interview with the TTP while inpatient. Patient demographics are presented in Table 1. On average, participants were middle aged (m = 50.0). Slightly over half were male (53.5%), and a little under a third were Black/African American (30.9%) with approximately half identifying as White (53.9). A majority of patients endorsed daily smoking (79.9%), averaging about 11 cigarettes per day and a smoking history of approximately 29 years. With respect to dependence, 64.8% reported smoking their first cigarette withing 5 min of waking, 15.7% between 5 and 30 min, 7.2% between 31 and 60 min, and 12.3% later than 60 min.

**Table 1:** Patient demographics and smoking characteristics.

**Characteristic (*N* = 4,153)**	**M or N**	**SD or %**
Age	49.97	14.81
**Sex**		
Male	2,223	53.5%
Female	1,929	46.4%
**Race/Ethnicity**		
White	2,238	53.9%
Black/African American	1,283	30.9%
Hispanic	51	1.2%
American Indian/Alaska native	17	<1%
Asian	14	<1%
Mixed/ Other	9	<1%
**Smoking Behavior**		
Daily smoking	3,320	79.9%
Cigarettes per day	15.7	11.07
Years smoking	29.06	15.50
**Time to first cigarette**		
<5 min	1,900	45.8%
6–30 min	484	11.7%
31–60 min	223	5.4%
>61 min	381	9.2%

Patient mental health diagnoses can be seen in Table 2. Overall, 34% of the patient sample was diagnosed with at least one mental health disorder. Within this subset of patients, the average number of diagnoses was 1.55 (SD = 0.89). The most common disorders diagnosed were Depression (11.1%), Alcohol Use Disorder (10.8%), Substance Use Disorder (8.5%), Anxiety (8.1%), and Chronic Pain (8.1%). Demographics of those with a mental health diagnosis did not differ significantly from those of the total sample. Of the 4,153 patients identified, follow-up data were available for 26% of patients (22.9% for those with mental health diagnoses, 27.7% for those without).

**Table 2:** Patient mental health diagnoses.

**Characteristic (*N* = 4,153)**	***N***	**%**
All mental health	1,417	34.1%
Depression	460	11.1%
Anxiety	335	8.1%
Chronic pain	336	8.1%
PTSD	58	1.4%
Alcohol use disorder	447	10.8%
Substance use disorder	353	8.5%
Serious mental illness	173	4.2%
Personality disorder	18	<1%
Childhood developmental disorder	16	<1%

### Mental Health and Tobacco Outcomes

Logistic regression analysis was utilized to investigate the effect of the presence of a mental health diagnosis on 7-day self-reported abstinence at 30-days post-discharge (ITT). Mental health diagnosis was found to significantly decrease the odds of abstinence at follow-up (B = 0.431, SE = 0.109, Wald = 15.69, *p* < 0.01). Those without a mental health diagnosis were significantly more likely to report abstinence at follow up (OR = 1.54; CI = 1.24–1.91) than those with a history of mental health diagnosis.

A second regression tested the effect of total number of mental health diagnoses on cigarettes per day at the time of intervention. Results of this analysis were also significant (B = 0.653, SE = 0.206, *t* = 3.17, *p* < 0.01) indicating that number of cigarettes smoked increases as the number of mental health conditions increase.

Finally, a series of Pearson correlations were conducted in order to characterize variables associated with smoking cessation with mental health problems. Results of these correlations can be seen in Table 3. Number of mental health diagnoses was associated with lower ratings of importance to quit (*r* = −0.04; *p* < 0.01) and lower self- efficacy with respect to quitting (*r* = −0.06; *p* < 0.01), but not associated with the number of quit attempts in the past year (*r* = −0.002; *p* = 0.912) or duration of most recent quit attempt (*r* = −0.009; *p* = 0.597). The mean reported values for importance to quit and self-efficacy to quit are reported in Table 4.

**Table 3:** Mental health diagnosis and quit attempt variables.

**Variable**	***n***	***M***	***SD***	**1**	**2**	**3**	**4**	**5**
1. Total mental health diagnoses	4,153	0.53	0.90	—				
2. Number of quit attempts in the past year	3,923	1.44	2.96	−0.002	—			
3. Importance to quit	4,128	3.84	1.33	−0.04^**^	0.15^**^	—		
4. Self-efficacy to quit	4,128	3.56	1.29	−0.06^**^	0.13^**^	0.75^**^	—	
5. Last quit duration	3,608	93.71	172.53	−0.009	0.22^**^	0.17^**^	0.21^**^	—

** *Indicates p < 0.01*.

**Table 4:** Importance to quit and self-efficacy to quit by mental health diagnosis category.

**Diagnostic category**	***N***	**Mean self-efficacy (Std. Dev.)**	**Mean importance to quit (Std. Dev.)**
Those without mental health diagnoses	2,736	3.58 (1.31)	3.85 (1.28)
Chronic pain	336	3.44 (1.35)	3.74 (1.38)
Anxiety	335	3.36 (1.33)	3.70 (1.37)
Alcohol use disorder	447	3.36 (1.31)	3.59 (1.42)
PTSD	58	3.32 (1.35)	3.62 (1.41)
Childhood dev. disorder	16	3.32 (1.29)	3.45 (1.41)
Depression	460	3.30 (1.33)	3.63 (1.40)
Substance use disorder	353	3.20 (1.32)	3.45 (1.40)
SMI	173	3.06 (1.36)	3.30 (1.50)
Personality disorder	18	2.80 (1.29)	3.17 (1.43)

## Discussion

This study analyzes the effect of mental health diagnoses on outcomes of a brief, opt-out tobacco treatment intervention among a general hospitalized population. This study has several strengths: (1) the results reflect data on a relatively large number of patients treated within the opt-out program, (2) data reflect the effectiveness of an established and practical intervention, (3) data reflect an important population in need of additional research. Rates of mental health conditions, such as depression and substance use disorders, were consistent with general inpatient hospital mental health diagnosis prevalence (20). Results of the present analysis indicate that mental health diagnoses are associated with increases in cigarettes per day. Additionally, patients with one or more mental health diagnoses are less likely to report abstinence from smoking following a brief inpatient tobacco cessation intervention than those without such a diagnosis. There are several well-documented barriers to smoking cessation for those with mental health conditions. Tobacco may represent an individual's attempt to cope with stress or negative emotion (21). Smoking has also been associated with neurobiological mediators impacting mental illness (22). Mental health conditions may also impact self-efficacy for behavior change, which is consistent with our results that showed lower self-efficacy and importance for quitting among patients with mental health diagnoses. These findings highlight an increased importance to engage in more tailored or intensive treatment. For example, incorporating opt-out referrals for follow-up and emphasizing elements of treatment shown to impact on co-morbid psychopathology (23, 24). However, mental health conditions were not associated with differences in quit attempts over the past year, indicating that these individuals are equally motivated to quit. Hospital-based opt-out interventions represent an important venue and opportunity to engage this population. Indeed, hospitals appear to be a venue in which a relatively large number of people who smoke and have mental health conditions appear, and as such we should be investigating tobacco treatment interventions with this population in hospitals.

### Limitations

There are several limitations of the present analysis that should be taken into consideration. No non-treatment control group was used for reference, which limits interpretations of tobacco cessation behavior in those with psychopathology with respect to baseline trends. Additionally, the large amount of missing data from follow-up may indicate responder bias.

### Public Health Implications

While brief hospital-based opt-out interventions have been shown to be effective and capable of reaching a large proportion of hospital patients, it is important to identify subgroups for whom more tailored treatment would be beneficial. Those with mental health conditions represent a large subset of general hospital patients, report heavier daily smoking, and are less likely to report abstinence following standard intervention. Tobacco treatment programs should work to develop and test treatments which better identify, characterize, and serve these patients.

## Data Availability Statement

The original contributions presented in the study are included in the article/supplementary materials, further inquiries can be directed to the corresponding author.

## Ethics Statement

The studies involving human participants were reviewed and approved by IRB-II - Medical University of South Carolina; ID - Pro00105610. Written informed consent for participation was not required for this study in accordance with the national legislation and the institutional requirements.

## Author Contributions

BS contributed conceptualization, methodology, data analysis writing–original draft, and writing–review and editing. AR and KC contributed methodology and writing–review and editing. AP, MF, and SS contributed to writing–review and editing. BT: conceptualization, methodology, and writing-review and editing. All authors contributed to the article and approved the submitted version.

## Funding

AR, BS, and BT report funding from the following NCI grants: R01 CA235697, R01 CA261232, R01 CA207229, and K07CA214839. AP is funded though NIH Institutional Postdoctoral Training Grant NIH-T32-HL144470.

## Conflict of Interest

BT and KC have both served as paid expert witnesses in litigation against cigarette manufacturers. BT also has received payment from Pfizer Inc., to serve on an Advisory Board exploring the role of e-cigarettes on smoking behavior. The remaining authors declare that the research was conducted in the absence of any commercial or financial relationships that could be construed as a potential conflict of interest.

## Publisher's Note

All claims expressed in this article are solely those of the authors and do not necessarily represent those of their affiliated organizations, or those of the publisher, the editors and the reviewers. Any product that may be evaluated in this article, or claim that may be made by its manufacturer, is not guaranteed or endorsed by the publisher.
